# P-184. Helminth co-infection in leprosy: Innocent bystander or immune dysregulator?

**DOI:** 10.1093/ofid/ofaf695.407

**Published:** 2026-01-11

**Authors:** Jessica K Fairley, Santino Diaz-Palma, Heloine Leite, Maisa Vieira, Lorena B P Oliveira, Pedro H F Marçal, Audra Bass, Marcos D S Pinheiro, Erica B M Silva, Julie A Clennon, Thomas R Ziegler, Lance A Waller, José A Ferreira, Jeffrey M Collins, Lucia A Fraga

**Affiliations:** Emory University, Division of Infectious Diseases, Atlanta, Georgia; Emory University Rollins School of Public Health, Atlanta, Georgia; Universidad Federal de Juiz de Fora-Campus Gov Valadares, Governador Valadares, Minas Gerais, Brazil; Univale University, Governador Valadares, Minas Gerais, Brazil; Univale University, Governador Valadares, Minas Gerais, Brazil; Georgia Institute of Technology, Atlanta, Georgia; Emory University, Atlanta, Georgia; Universidade Federal De Juiz De Fora, Governador Valadares, Minas Gerais, Brazil; Universidade Federal de Juiz de Fora- Campus Gov Valadares, Governador Valadares, Minas Gerais, Brazil; Emory University College of Arts and Sciences, Atlanta, Georgia; Emory University School of Medicine, Atlanta, Georgia; Emory University, Atlanta, Georgia; Unifenas University, Belo Horizonte, Minas Gerais, Brazil; Emory University School of Medicine, Atlanta, Georgia; Universidade Federal de Juiz de Fora - Campus GV, Governador Valadares, Minas Gerais, Brazil

## Abstract

**Background:**

Leprosy, a neglected tropical disease also called Hansen’s disease, remains a public health challenge in Brazil. Helminth infections have been shown to be associated with leprosy, but results have been mixed. We present preliminary results from a longitudinal cohort to address this question.

**Figure 1.**
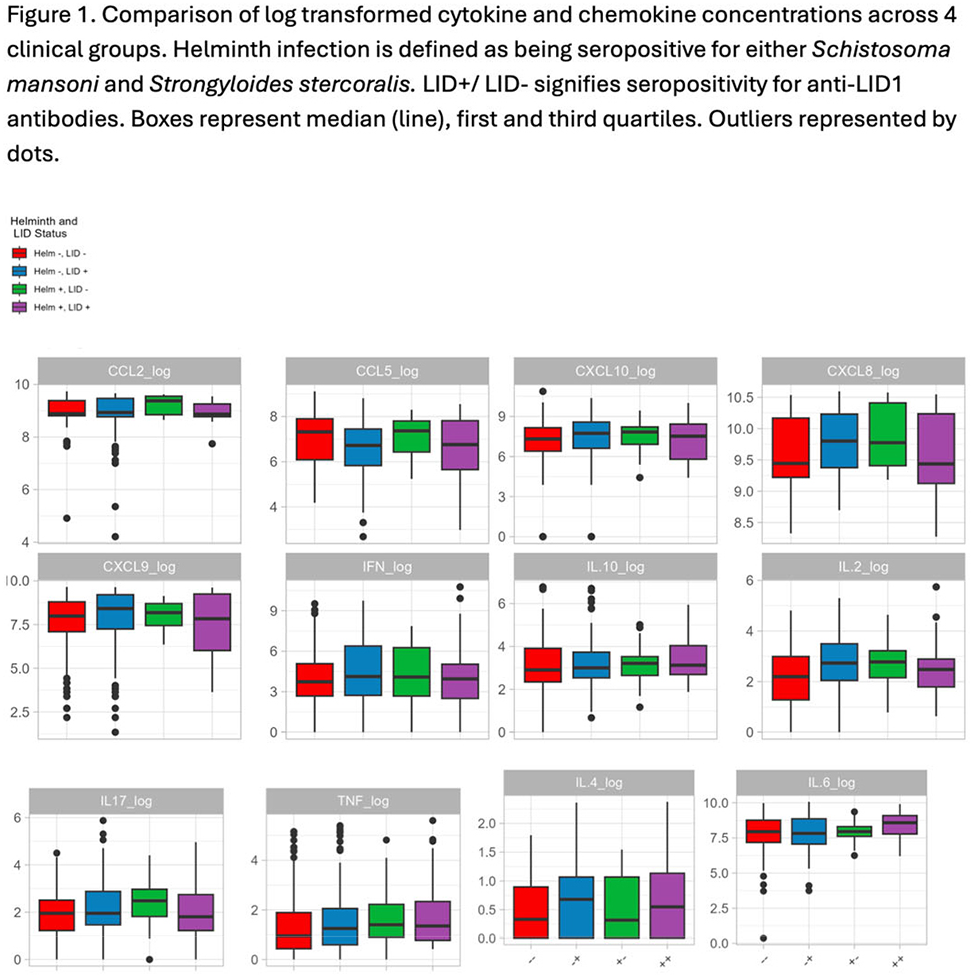
Cytokine and chemokine comparisons across infection groups. Comparison of log transformed cytokine and chemokine concentrations across 4 clinical groups. Helminth infection is defined as being seropositive for either Schistosoma mansoni and Strongyloides stercoralis. LID+/ LID- signifies seropositivity for anti-LID1 antibodies. Boxes represent median (line), first and third quartiles. Outliers represented by dots.

**Methods:**

Individuals ages 3 years and up (n=1315) were enrolled in eastern Minas Gerais, Brazil, and screened for anti-LID1, a *Mycobacterium leprae* specific antibody. Those positive without a history of leprosy and anti-LID1 negative controls were enrolled in the longitudinal study and evaluated for leprosy over time. Helminth serology (*Schistosoma mansoni* (SM) and *Strongyloides stercoralis* (SS*)*) by multiplexed beaded assay and stool helminth exams were performed at the onset. We then measured cytokines and chemokines from PBMC stimulated by *M. leprae* (ML) antigen at time 0.

**Results:**

Seventy-seven (6%) individuals tested positive for anti-LID1. Of the longitudinal cohort (n = 153), 17 (11%) were seropositive for SM and 11 (7%) for SS. One individual tested positive for SM by stool. Twenty (26%) anti-LID1+ individuals were diagnosed with leprosy, half at the initial visit. Seropositivity for either SM or SS was associated with a lower likelihood of leprosy (RR = 0.83, 95% CI 0.69, 0.99) in anti-LID1+ individuals. Median concentrations of CXCL8 (p=0.07) were lower in SM-ML seropositive individuals compared to anti-LID1+ alone, and IL17, IL2, IL6 showed interesting trends in co-infection (Figure 1).

**Conclusion:**

While helminths have not been associated with risk of leprosy in our cohort to date, the immune findings suggest a potential alteration of cytokine / chemokine response in co-infected (or co-seropositive) individuals. Interestingly, we found similar results with CXCL8 and IL17 in a previous study on co-infection, highlighting a potential mechanistic pathway that should be further studied.

**Disclosures:**

All Authors: No reported disclosures

